# Temporal Changes in Fetal and Maternal Parameters in Early‐Onset Fetal Growth Restriction: A Multicenter, Retrospective Cohort Study

**DOI:** 10.1111/1471-0528.70073

**Published:** 2025-11-03

**Authors:** Mette van de Meent, Ewoud Schuit, Wessel Ganzevoort, Salwan Al‐Nasiry, Mireille N. Bekker, Jan B. Derks, Johannes J. Duvekot, Sanne J. Gordijn, Floris Groenendaal, Reint Jellema, H. Marieke Knol, Elisabeth M. W. Kooi, René F. Kornelisse, Gwendolyn T. R. Manten, Susanne M. Mulder‐De Tollenaer, Wes Onland, Eline van der Wilk, Hans Wolf, A. Titia Lely, Judith Kooiman

**Affiliations:** ^1^ Department of Obstetrics and Gynaecology Wilhelmina Children's Hospital, University Medical Center Utrecht and Utrecht University Utrecht the Netherlands; ^2^ Julius Center for Health Sciences and Primary Care University Medical Center Utrecht, Utrecht University Utrecht the Netherlands; ^3^ Department of Obstetrics and Gynaecology Amsterdam University Medical Centers, Location AMC Amsterdam the Netherlands; ^4^ Amsterdam Reproduction and Development Research Institute Amsterdam the Netherlands; ^5^ Department of Obstetrics and Gynaecology Maastricht University Medical Center Maastricht the Netherlands; ^6^ Department of Obstetrics and Gynaecology Erasmus Medical Center Rotterdam the Netherlands; ^7^ Department of Obstetrics and Gynaecology University Medical Center Groningen, University of Groningen Groningen the Netherlands; ^8^ Department of Neonatology, Wilhelmina Children's Hospital University Medical Center Utrecht and Utrecht University Utrecht the Netherlands; ^9^ Department of Neonatology Maastricht University Medical Center Maastricht the Netherlands; ^10^ Department of Obstetrics and Gynaecology Isala Zwolle the Netherlands; ^11^ Division of Neonatology, Beatrix Children's Hospital University Medical Center Groningen, University of Groningen Groningen the Netherlands; ^12^ Department of Neonatal and Paediatric Intensive Care, Division of Neonatology Erasmus Medical Center Rotterdam the Netherlands; ^13^ Department of Neonatology Isala Zwolle the Netherlands; ^14^ Department of Neonatology Amsterdam University Medical Centers Location AMC Amsterdam the Netherlands

**Keywords:** cardiotocography, doppler, early‐onset fetal growth restriction, short‐term variability, time sequence

## Abstract

**Objective(s):**

Timing of birth is complex in early‐onset fetal growth restriction (FGR) and the literature is limited regarding the exact sequence of changes in antenatal parameters. This study aimed to examine this sequence in a large early‐onset FGR cohort.

**Design:**

Multicenter, retrospective cohort study.

**Setting:**

Six tertiary care hospitals in the Netherlands.

**Population:**

A post hoc analysis of the OPtimal TIming of antenatal COrticosteroids in early‐onset fetal growth REstriction (OPTICORE) study was performed.

**Methods:**

Repeated measures of antenatal parameters were assessed routinely from diagnosis to birth. Mixed‐effects models were used to determine the probability of abnormality (binary) and the trend (Z‐score, continuous) over time for each parameter (< 32 and ≥ 32 weeks).

**Main Outcome Measures:**

The time sequence of changes in fetal and maternal health parameters from diagnosis to birth in early‐onset FGR pregnancies.

**Results:**

A total of 1453 patients were included, of whom 1025 patients gave birth < 32 weeks and 428 ≥ 32 weeks, with median gestational ages of 29 + 3 weeks (IQR 27 + 6, 30 + 5) and 34 + 4 weeks (IQR 33 + 0, 37 + 0), respectively. The most apparent changes in fetal parameters in the last days preceding birth comprised: absent/reversed end‐diastolic velocity of the umbilical artery and cardiotocography abnormalities. Regarding maternal parameters, an increased probability of antihypertensive agent(s) use was seen shortly preceding birth < 32 weeks.

**Conclusion(s):**

This large cohort of early‐onset FGR pregnancies provides a time sequence of fetal and maternal health parameters from diagnosis to birth, which could inform clinical management.

**Abbreviations**
ACS: antenatal corticosteroids
CTG: cardiotocography
FGR: fetal growth restriction
OPTICORE: OPtimal TIming of antenatal COrticosteroids in pregnancies complicated by early‐onset fetal growth REstriction
SD: standard deviation
STV: short‐term variability

## 1 Introduction

Early‐onset fetal growth restriction (FGR) is defined as failure of a fetus to reach its growth potential with its diagnosis before 32 weeks of pregnancy [1]. It occurs in 0.5%–1.0% of all pregnancies and is an important cause of neonatal morbidity (24%) and mortality (8%–19%) [2, 3, 4, 5]. In high‐income countries, it is most commonly caused by placental insufficiency leading to hypoxic circumstances in utero [6, 7]. To prevent the fetus from sequelae from hypoxia, early‐onset FGR frequently requires iatrogenic, preterm birth.

Timing of birth is complex in pregnancies complicated by early‐onset FGR, as physicians have to weigh the risks of preterm birth against the risks of hypoxic damage including stillbirth or severe neurological damage. Additionally, concomitant (pre‐)eclampsia is diagnosed in 30%–40% of these pregnancies, which could also require preterm birth [6, 8, 9]. Since the timing of birth is complex, so is determining the optimal management of early‐onset FGR. Despite the performance of landmark trials, significant practice variation exists in the management of early‐onset FGR across and within countries [3, 4, 10]. This is likely due to gaps in knowledge on the optimal method and frequency of fetal surveillance along with an ongoing debate on the timing and ideal diagnostic test to instigate preterm birth.

Understanding the chronological progression of maternal and fetal parameters between FGR diagnosis and birth could guide clinical decision‐making, for example, in timing of antenatal corticosteroids (ACS) and to determine monitoring setting and frequency, and whether a patient needs to be transferred to a level‐III hospital. Hecher et al. described the time sequence of changes in fetal parameters in a small cohort of 110 pregnancies complicated by FGR, but maternal parameters were not included [11]. Furthermore, within the population of early‐onset FGR different levels of severity and scenarios of disease progression exist. Therefore, identifying distinct early‐onset FGR subgroups could further aid clinical management.

The main objective of this study was to describe the time sequence of changes in fetal and maternal parameters in a large contemporary cohort of early‐onset FGR pregnancies. Second, this study aimed to link predefined subgroups of early‐onset FGR pregnancies to neonatal outcomes to further guide clinical decision‐making.

## 2 Materials and Methods

### 2.1 Study Design

This study was a post hoc analysis of the data from the OPtimal TIming of antenatal COrticosteroids pregnancies complicated by early‐onset fetal growth REstriction (OPTICORE‐) cohort, which aimed to optimise the timing of ACS administration in early‐onset FGR. Methods of the OPTICORE study have been published more extensively previously [12]. In short, a multicenter, retrospective cohort study was performed in six tertiary teaching hospitals in the Netherlands between 2012 and 2021. To be eligible for inclusion within the OPTICORE cohort patients had to be diagnosed with early‐onset FGR in accordance with the consensus‐based definition of Gordijn et al. [13], it had to be a singleton pregnancy, patients had to opt for active fetal management and they had to be ≥ 18 years of age. Multiple pregnancies, pregnancies diagnosed with a fetal genetic or congenital disorder and patients who indicated that their data or offspring data were not available for scientific purposes were excluded. Within the OPTICORE study, repeated measurements were included on the day of each ultrasound examination. Monitoring strategies varied across the six participating hospitals and were based on local protocols. In hospitals following an early hospital admission strategy (Utrecht, Rotterdam, Zwolle), patients were admitted once an abnormal pulsatility index of the umbilical artery was detected and monitored daily with cardiotocography, weekly with Doppler measurements of the umbilical and middle cerebral arteries, and biweekly with growth scans. In hospitals following a late hospital admission strategy (Amsterdam, Maastricht, Groningen), admission occurred only after absent or reversed end‐diastolic velocity in the umbilical artery was detected. In both strategies, absent/reversed end‐diastolic velocity led to intensified monitoring: twice‐daily CTG, twice‐weekly Doppler measurements of the umbilical artery and middle cerebral artery, and biweekly growth scans. Results of this study were reported in line with the STrengthening the Reporting of OBservational studies in Epidemiology (STROBE) statement (File S1) [14]. Patients were not involved in the design of this study.

### 2.2 Collection of Parameters

Fetal and maternal parameters were collected routinely from the diagnosis of early‐onset FGR until birth. Variables were selected based on their routine use in clinical practice for the monitoring and management of early‐onset FGR, in line with clinical management guidelines [15, 16, 17]. The frequency of monitoring and availability of parameters depended on the severity of the disease in accordance with local monitoring guidelines. Fetal parameters comprised ultrasound parameters (i.e., pulsatility index of the umbilical artery and the middle cerebral artery; abnormality based on Baschat [18], Parra‐Cordero [19] or Harrington [20]), the cerebroplacental ratio and the pulsatility index of veins of the ductus venosus (abnormality based on Hecher 1994), cardiotocography (CTG) assessment as performed by local obstetricians based on the Federation of Gynaecology and Obstetrics consensus guidelines on intrapartum fetal monitoring and short‐term variability (STV) [11, 18, 19, 20, 21]. STV was retrospectively determined if raw digital CTG data files were retrievable, which was the case for the majority of patients except for patients from one hospital responsible for the lowest inclusions. Thresholds for abnormality for fetal parameters were summarised in Table S1, and were based on local charts. Maternal parameters comprised the use of antihypertensive agents (oral or intravenous), diagnosis of pre‐eclampsia or hemolysis elevated liver enzymes low platelets (HELLP) syndrome, and use of magnesium sulfate on maternal indication.

### 2.3 Subgroups of Early‐Onset FGR Pregnancies

Three main subgroups of early‐onset FGR pregnancies were distinguished: (1) early‐onset FGR based on biometric values only, with normal pulsatility indices of the umbilical artery (i.e., < 95th centile); (2) early‐onset FGR with abnormal pulsatility indices of the umbilical artery (i.e., > 95th centile) with positive end‐diastolic velocity; and (3) early‐onset FGR with at least two instances of absent or reversed end‐diastolic velocity. Subsequently, these three subgroups were subdivided in patients with and without a diagnosis of pre‐eclampsia. The subgroups were based on expert opinion and subdivided in patients with and without concomitant pre‐eclampsia. For each subgroup, gestational age at birth, birthweight, rates of prelabour caesarean section, rates of birth < 32 weeks and a composite of adverse perinatal outcomes (i.e., perinatal or in‐hospital mortality, necrotizing enterocolitis ≥ 2A, moderate or severe bronchopulmonary dysplasia, cystic periventricular leukomalacia, intraventricular haemorrhage grade 3 or venous infarction and/or culture‐proven sepsis) were described [3].

### 2.4 Statistical Analysis

Baseline characteristics were summarised as mean with standard deviation (SD), median with interquartile range (IQR) or number with percentages (%), as appropriate. Repeated measures of fetal and maternal parameters were analysed with a multilevel model using the ‘lme’ package to determine the association between the time difference in days before birth and the probability of abnormality (i.e., in a binary way). Furthermore, for the pulsatility index of the umbilical artery and the middle cerebral artery, the pulsatility index of veins of the ductus venosus and the STV the association between the time difference and the trend over time was assessed continuously. To standardise values relative to the expected mean for a given gestational age and indicate how many standard deviations a measurement deviates from the mean, Z‐scores were calculated using reference charts for each of the measurements for this continuous trend over time [19, 20, 22]. A random intercept and/or slope was added, as appropriate, per patient to adjust for multiple measurements within one patient. Analyses were stratified according to gestational age at birth (i.e., < 32 and ≥ 32 weeks). The reference for this analysis was defined as the time of birth, which was indicated by the attending physician having a significant suspicion of developing fetal hypoxia, typically because of repeated unprovoked decelerations on the fetal heart rate tracing, or indicated by severe maternal disease (in accordance with Hecher et al. [11]). As a sensitivity analysis, the binary analyses on the probability of abnormality were repeated in the patient group without concomitant pre‐eclampsia (defined as the presence of hypertension with proteinuria or other maternal organ dysfunction) [23]. Also, for the identified subgroups of early‐onset FGR pregnancies the binary analyses on the probability of abnormality for fetal and maternal parameters were repeated. The ‘ggplot2’ package was used subsequently to visualise these data.

## 3 Results

### 3.1 Baseline Characteristics

A total of 1453 patients were eligible for inclusion in this post hoc analysis of the OPTICORE study. Demographic and baseline characteristics have been summarised in Table 1. The mean birthweight was 919 g (SD 260) in the group that birthed < 32 weeks of pregnancy versus 1600 g (SD 391) in the group that birthed ≥ 32 weeks of pregnancy (Table 2). Median gestational ages at birth were 29 + 3 weeks (IQR 27 + 6, 30 + 5) and 34 + 4 weeks (IQR 33 + 0, 37 + 0), respectively. The total number of patients along with the number of measurements for each parameter has been provided in Table S2.

**TABLE 1:** Demographic and baseline characteristics.

	Birth < 32 weeks (*N* = 1025)	Birth ≥ 32 weeks (*N* = 428)
Demographic		
Age	30.4 (5.4)	31.1 (5.4)
BMI	26.3 (6.2)	25.2 (6.1)
Caucasian ethnicity	640 (62.4)	265 (61.9)
Medical history		
Chronic hypertension	162 (15.8)	41 (9.6)
Chronic kidney disease	33 (3.3)	14 (3.2)
Pre‐existent diabetes mellitus	23 (2.3)	9 (2.1)
Smoking during pregnancy	141 (13.8)	86 (20.1)
Obstetric history		
Previous pregnancy	343 (33.5)	182 (42.5)
FGR	101 (9.9)	54 (12.6)
PIH	29 (2.8)	7 (1.6)
PE/HELLP	95 (9.3)	23 (5.4)
Preterm birth	145 (14.1)	61 (14.3)
Perinatal mortality	42 (4.1)	23 (5.4)
Diabetes gravidarum	16 (1.6)	5 (1.2)
Current pregnancy		
PIH	232 (22.6)	97 (22.7)
PE/HELLP	644 (62.8)	145 (33.9)
Medication use in current pregnancy		
Use of antihypertensive agents	666 (65.0)	189 (44.2)
Use of acetylsalicylic acid	128 (12.5)	58 (13.6)
Use of magnesium sulphate	727 (70.9)	94 (22.0)
Administration of ACS	1002 (97.8)	295 (68.9)

*Note:* Data provided as mean (SD), median (IQR) or *N* (%) as appropriate.

Abbreviations: ACS, antenatal corticosteroids; BMI, body mass index; HELLP, hemolysis elevated liver enzymes low platelets; PE, pre‐eclampsia; PIH, pregnancy‐induced hypertension.

**TABLE 2:** Birth and neonatal outcome data.

	Birth < 32 weeks (*N* = 1025)	Birth ≥ 32 weeks (*N* = 428)
Birth		
Start of birth		
Spontaneous	53 (5.2)	42 (9.8)
Iatrogenic	972 (94.8)	386 (90.2)
Mode of birth		
Vaginally	51 (5.0)	137 (32.0)
Prelabour caesarean section	937 (91.4)	217 (50.7)
Emergency section	37 (3.6)	74 (17.3)
Neonatal outcomes		
Gestational age at birth (weeks)	29 + 3 (27 + 6, 30 + 5)	34 + 4 (33 + 0, 37 + 0)
Birthweight (grams)	919 (260)	1600 (391)
Birthweight centile	1.44 (0.9)	1.25 (0.7)
Male sex	488 (47.6)	179 (41.8)

*Note:* Data provided as mean (SD), median (IQR) or *N* (%) as appropriate.

### 3.2 Fetal Parameters

**FIGURE 1:**
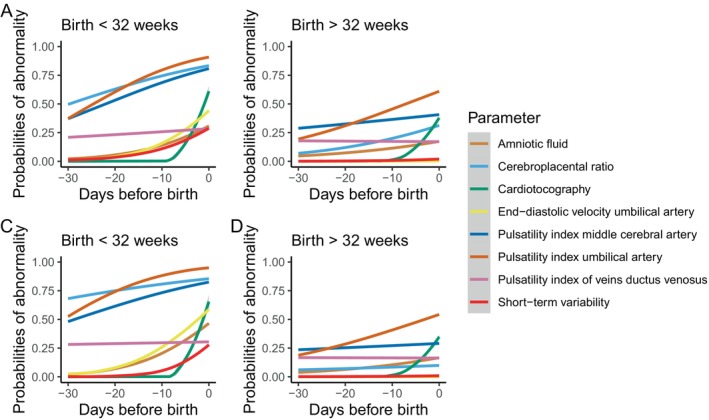
Time sequence of fetal parameters in relation to time before birth stratified according to gestational age at birth. (A,B) describe the time sequence of parameters in the complete group of early‐onset FGR pregnancies, (C and D) describe the time sequence in patients with early‐onset FGR without concomitant PE.

**FIGURE 2:**
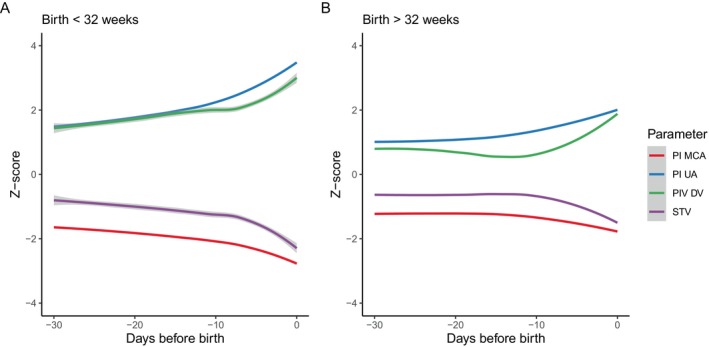
Trend of fetal parameters (continuous) in relation to time before birth stratified according to gestational age at birth. DV, ductus venosus; MCA, middle cerebral artery; PI, pulsatility index; PIV, pulsatility index of veins; STV, short‐term variability.

The time sequence of the probability of abnormality regarding fetal parameters in relation to time before birth has been depicted in Figure 1. As can be appreciated from Figure 1, the cerebroplacental ratio and the pulsatility index of the umbilical artery and the middle cerebral artery had a higher probability of becoming abnormal first in pregnancies with a birth before 32 weeks of gestation. In this population, the last days preceding birth the end‐diastolic velocity in the umbilical artery and the cardiotocography trace had a steep increase in the probability of abnormality (i.e., absent/reversed end‐diastolic velocity), with probabilities of abnormality increasing from 25% to 50% and 10% to 55% starting 7 days prior to birth, respectively. The most important fetal health parameter indicative of upcoming birth was the occurrence of CTG abnormalities. For patients with early‐onset FGR who birthed after 32 weeks of gestation, the pulsatility index of the middle cerebral artery had a higher probability of becoming abnormal first, followed by the pulsatility index of the umbilical artery. Similar to the patients who birthed before 32 weeks, the visual CTG evaluation became abnormal in the last days preceding birth, although this was in a much smaller proportion of patients. Results of the sensitivity analyses in the group without concomitant pre‐eclampsia are provided in Figure 1C,D, showing similar results. Furthermore, fetal parameters have also been assessed in a continuous way (Z‐scores) to determine the trend over time, of which results have been provided in Figure 2, showing similar changes over time (i.e., first the pulsatility index of the umbilical artery and middle cerebral artery tend to become abnormal, followed by the STV as well as the ductus venosus in the last days preceding birth). To add, Figure 2 shows that the pulsatility index of the umbilical artery and the middle cerebral artery tend to become further abnormal (i.e., with increased Z‐scores) in the last days prior to birth.

### 3.3 Maternal Parameters

**FIGURE 3:**
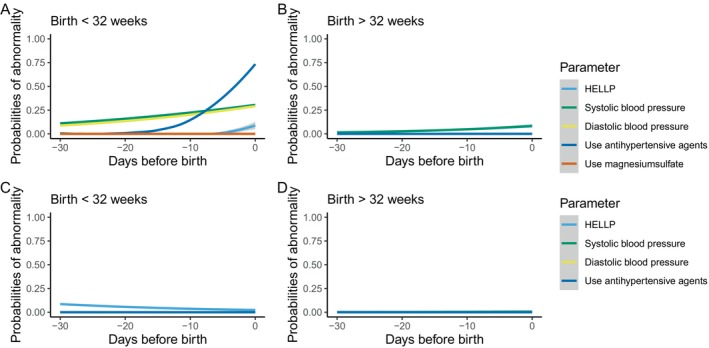
Time sequence of maternal parameters in relation to time before birth stratified according to gestational age at birth. HELLP, hemolysis elevated liver enzymes low platelets. (A,B) describe the time sequence of parameters in the complete group of early‐onset FGR pregnancies, (C and D) describe the time sequence in patients with early‐onset FGR without concomitant PE.

Figure 3A–D reflect the time sequence of maternal parameters in relation to time before birth. Remarkably, maternal parameters became solely abnormal in the group including patients with pre‐eclampsia who birthed before 32 weeks of gestation (Figure 3A,B). In this group, the last days preceding birth the probability of use of antihypertensive agent was increased to almost 75%.

### 3.4 Subgroups of Early‐Onset FGR Pregnancies

In Table 3 birth and neonatal outcomes were provided for each early‐onset FGR subgroup. Gestational age at birth and birthweight were lower with increasing severity of early‐onset FGR. Furthermore, rates of prelabour caesarean section (100% vs. 44.2%), rates of birth < 32 weeks (86.3% vs. 42.3%) and the adverse composite perinatal outcome (47.5% vs. 19.7%) were increased in patients with the most severe type of early‐onset FGR as compared to the least severe type of early‐onset FGR. Regarding the time sequence of changes in fetal and maternal parameters in relation to the time preceding birth for each subgroup, the time sequences of fetal parameters, in particular, show increased probabilities of abnormality with increasing severity of the FGR phenotype (Figures S1 and S2).

**TABLE 3:** Subgroups of early‐onset FGR patients.

Subgroups	Diagnosis PE	Number of patients	Prelabour caesarean section	GA at birth (weeks)	Birthweight (grams)	Birth < 32 weeks	Adverse composite
1. Early‐onset FGR with only normal PIs in the umbilical artery (i.e., < 95th centile)	No	142	63 (44.2)	32 + 5 (30 + 2, 37 + 0)	1526 (511)	60 (42.3)	28 (19.7)
	Yes	161	129 (80.1)	30 + 4 (28 + 4, 31 + 5)	1115 (341)	127 (78.9)	52 (32.3)
2. Early‐onset FGR with abnormal PI of the UA (i.e., > 95th centile) with solely positive EDF	No	183	107 (58.5)	33 + 3 (30 + 3, 36 + 5)	1385 (515)	76 (41.5)	36 (19.7)
	Yes	265	233 (87.9)	30 + 5 (2 + 3)	1099 (291)	201 (75.8)	88 (33.2)
3. Early‐onset FGR with at least two times A/REDF	No	162	150 (92.6)	29 + 6 (28 + 1, 31 + 5)	845 (661, 1094)	125 (77.2)	79 (48.8)
	Yes	139	139 (100.0)	29 + 3 (2 + 2)	880 (275)	120 (86.3)	66 (47.5)

*Note:* Values have been provided as number (*N*) with percentages, median with interquartile ranges or mean with standard deviation as appropriate. The adverse composite outcome was defined as one or more of the following outcome measures: perinatal and in‐hospital mortality, intraventricular haemorrhage grade 3 or venous infarction, moderate or severe bronchopulmonary dysplasia, culture‐proven sepsis, necrotizing enterocolitis Bell's stage ≥ 2A, cystic periventricular leukomalacia. A total of 184 patients were not assigned to any of the three subgroups.

## 4 Discussion

### 4.1 Main Findings

This study provides the time sequences of changes in fetal and maternal parameters in relation to time before birth in early‐onset FGR pregnancies. The last days preceding birth, the end‐diastolic velocity in the umbilical artery and visual CTG assessment had an increased probability of becoming abnormal. Regarding maternal parameters, there was an increased risk of the use of antihypertensive agent(s) the last days preceding birth (birth < 32 weeks). Furthermore, we observed a clear association between the predefined early‐onset FGR subgroups and neonatal outcomes, which could make this subdivision useful for clinical management.

### 4.2 Interpretation

The time sequence of changes in parameters provided by our study is compliant with the time sequence provided by Hecher et al. and Ferrazzi et al. [11, 24]. Nevertheless, the probability of abnormality for the umbilical artery pulsatility index, the pulsatility index of veins of the ductus venosus, the end‐diastolic velocity (Ferrazzi), and the amniotic fluid index (Hecher) seems to be higher in the time sequence provided by their studies (e.g., > 80% versus 60% in our study for the umbilical artery pulsatility index at 15 days before birth in the group with birth < 32 weeks) [11]. Differences in these probabilities can possibly be explained by the: (1) use of different definitions of early‐onset FGR (fetal abdominal circumference < 5th centile for gestational age with or without pregnancy‐induced hypertension (Hecher) or fetal abdominal circumference < 2nd centile for gestational age with an abnormal pulsatility index of the umbilical artery (Ferrazzi) in their studies versus the consensus‐based definition of Gordijn et al. in our study); (2) use of different statistical models (logistic regression versus a multilevel model); (3) fact that the last measurement had to be < 24 h before birth in the study of Hecher et al., while this was not a criterion in our study; or (4) fact that their study was performed > 20 years ago and detection and management of early‐onset FGR pregnancies possibly changed over time, for example, in terms of timing of birth leading to different probabilities of abnormality of parameters prior to birth [11]. To our knowledge, there is limited prior work on the link between distinct subgroups of early‐onset FGR and neonatal outcomes. Results of this analysis align with previous literature on increased rates of adverse perinatal outcomes in cases with absent or reversed end‐diastolic velocity [25, 26, 27].

### 4.3 Clinical Implications

No consensus exists on monitoring frequency and setting, timing of ACS and the diagnostic trigger to instigate preterm birth in early‐onset FGR, despite the performance of two landmark trials and international guideline recommendations [1, 3, 10, 15]. The progression of fetal and maternal health parameters along with distinct subgroups of early‐onset FGR pregnancies could inform management in this setting. For instance, this information could aid physicians in defining: (1) whether patients should be referred to a level‐III hospital if birth is anticipated in the following days; (2) when to administer ACS as ACS seem to be most beneficial in reducing neonatal morbidity and mortality if administered within 1 week preceding birth [28]; (3) the monitoring setting and intensity. In early‐onset FGR patients with normal pulsatility indices of the umbilical artery and no diagnosis of pre‐eclampsia, less intensive outpatient monitoring might also be safe. This hypothesis should first be externally validated before firm statements can be made.

### 4.4 Research Implications

As birthweights and gestational ages at birth appear to decrease with the increasing severity of early‐onset FGR, and prelabour caesarean sections and adverse perinatal outcomes become more common, future research should aim to establish appropriate monitoring strategies for various subgroups of early‐onset FGR pregnancies. It is crucial to find a balance between fetal‐ and maternal safety, patient satisfaction, and cost‐effectiveness in determining the location (outpatient vs. inpatient) and frequency of assessments. Since this study analysed the behaviour of early‐onset FGR pregnancies as a group, there is still an urgent need for tools producing a patient‐tailored risk assessment for the time to (indicated) birth. Considering the fact that management of early‐onset FGR includes both fetal and maternal health, patient‐tailored management should be achieved by a multivariate approach.

### 4.5 Strengths and Limitations

Strengths of this study comprise (1) the size of the cohort with early‐onset FGR pregnancies (i.e., 1453 pregnancies), for which repeated measurements were gathered on every day of each ultrasound follow‐up (a total of *N* = 75 319), the risk of overfitting was minimised, supporting the robustness of the provided trend lines; (2) the use of the consensus‐based definition of early‐onset FGR, and (3) the use of a multilevel model to adjust for multiple measurements within one patient.

Limitations were that (1) due to feasibility reasons, measurements were only included on the day of every ultrasound scan, while for some measurements (e.g., cardiotocography, blood pressure) more measurements would have been available but were left out of the dataset. Inclusion of these daily measurements might have resulted in an even more precise time sequence for these parameters; (2) the predicted probabilities of CTG abnormalities might be biased by indication, since this was the trigger to indicate birth. However, this does reflect clinical practice, as international guidelines on FGR advise striving for birth when repeated decelerations on CTG occur; (3) the monitoring frequency differed between patients, mostly depending on disease severity, which could have influenced the accuracy of the time sequence further, although this also reflects current clinical practice; (4) due to regularisation, model predictions are pulled towards the mean for better generalizability, resulting in less extreme probabilities compared to observed values; (5) the ductus venosus and its pulsatility index of veins are not routinely measured in most centres in the Netherlands, and consequently, the time sequence of the ductus venosus might be less reliable due to a lower number of measurements; (6) the pulsatility index of the uterine artery is not consistently measured during pregnancy in early‐onset FGR and therefore it was not possible to provide the time sequence for this parameter; (7) although changes in the MCA PI seem to precede changes in the UA PI in pregnancies delivered after 32 weeks, it should be noted that this is a relatively small subset of the cohort and applies to a group with a relatively mild FGR phenotype—further investigation in larger cohorts would be needed to confirm its clinical relevance; (8) since the aim was descriptive rather than causal inference the provided models do not explicitly adjust for time‐dependent confounding.

## 5 Conclusion

This large cohort of recent early‐onset FGR pregnancies provides a time sequence of changes in fetal and maternal health parameters from diagnosis to birth and determines the prognosis for different subgroups of early‐onset FGR pregnancies. This valuable information could aid clinical management of these high‐risk pregnancies and highlights the need for a multivariate approach to achieve patient‐tailored management in early‐onset FGR.

## Author Contributions

M.M., W.G., A.T.L. and J.K. conceived and designed research; M.M., E.S. and J.K. analysed data; M.M., W.G., H.W., A.T.L. and J.K. interpreted results of analyses; M.M. and E.S. prepared figures; M.M. and J.K. drafted manuscript; M.M., W.G., S.J.G., E.K., W.O., H.W., J.B.D., E.W., R.F.K., S.A., R.J., H.K., G.T.R.M., S.M., J.B.D., F.G., M.N.B., E.S., A.T.L. and J.K. edited and revised manuscript. All authors approved the final version of the manuscript.

## Ethics Statement

The protocol of this study was assessed by the Ethics Committee of the University Medical Center Utrecht (METC NedMec, registration number 22/613), which confirmed that the Medical Research Involving Human Subjects Act (WMO) does not apply to this study.

## Consent

The authors have nothing to report.

## Conflicts of Interest

The authors declare no conflicts of interest.

## Supporting information


**Table S1:** Thresholds for abnormality of fetal parameters.
**Table S2:** Number of patients and measurements per parameter stratified according to gestational age at birth.
**Figure S1:** Time sequence of fetal parameters in relation to time before birth stratified according to gestational age at birth per subgroup.
**Figure S2:** Time sequence of maternal parameters in relation to time before birth stratified according to gestational age at birth per subgroup.
**File S1:** STROBE Statement—checklist of items that should be included in reports of observational studies.

## Data Availability

Data available on request from the authors.
