# Supplementary material for: Temporal Changes in Fetal and Maternal Parameters in Early‐Onset Fetal Growth Restriction: A Multicenter, Retrospective Cohort Study
Source: BJOG. 2025 Nov 3;133(4):671–9. doi: 10.1111/1471-0528.70073 (PMC12884221; doi:10.1111/1471-0528.70073)
Supplement: Supplementary file 1 — Table S1: Thresholds for abnormality of fetal parameters. Table S2: Number of patients and measurements per parameter stratified according to gestational age at birth. Figure S1: Time sequence of fetal parameters in relation to time before birth stratified according to gestational age at birth per subgroup. Figure S2: Time sequence of maternal parameters in relation to time before birth stratified according to gestational age at birth per subgroup. File S1: STROBE Statement—checklist of items that should be included in reports of observational studies. [file BJO-133-671-s001.docx]

**SUPPLEMENTARY INFORMATION**

**Table S1.** Thresholds for abnormality of fetal parameters

| Fetal parameter | Threshold for abnormality |
| --- | --- |
| Amniotic fluid | Oligohydramnios based on the single deepest pocket method (< 2 centimeter^1^ |
| Cardiotocography | Classification of suboptimal, abnormal or preterminal according to FIGO^2^ |
| Cerebroplacental ratio | <1.0 |
| End-diastolic velocity umbilical artery | Absent or reversed |
| Pulsatility index of middle cerebral artery | <5^th^ centile |
| Pulsatility index of umbilical artery | >95^th^ centile |
| Pulsatility index of veins ductus venosus | >95^th^ centile |
| Short-term variability | <3.5 until 29 weeks of gestation and <4.0 from 29 weeks of gestation onwards^3^ |

Abbreviations: cm, centimeter; FIGO, Federation of Gynecology and Obstetrics.

**Table S2.** Number of patients and measurements per parameter stratified according to gestational age at birth

| **Parameters** | **GA at birth** | **Number of patients** | **Number of measurements** |
| --- | --- | --- | --- |
| **Fetal** |  |  |  |
| PI UA | <32 weeks | 984 | 3137 |
|  | ≥32 weeks | 428 | 3784 |
| EDF | <32 weeks | 986 | 3232 |
|  | ≥32 weeks | 428 | 3748 |
| PI MCA | <32 weeks | 927 | 2862 |
|  | ≥32 weeks | 417 | 3139 |
| CPR | <32 weeks | 906 | 2816 |
|  | ≥32 weeks | 415 | 3175 |
| PIV DV | <32 weeks | 345 | 735 |
|  | ≥32 weeks | 158 | 454 |
| STV | <32 weeks | 591 | 1568 |
|  | ≥32 weeks | 270 | 1575 |
| Amnion fluid | <32 weeks | 982 | 2788 |
|  | ≥32 weeks | 425 | 3258 |
| CTG | <32 weeks | 1007 | 3036 |
|  | ≥32 weeks | 426 | 2921 |
|  |  |  |  |
| **Maternal** |  |  |  |
| SBP | <32 weeks | 947 | 2626 |
|  | ≥32 weeks | 420 | 3040 |
| DBP | <32 weeks | 947 | 2627 |
|  | ≥32 weeks | 420 | 3044 |
| HELLP diagnosis | <32 weeks | 1022 | 3327 |
|  | ≥32 weeks | 428 | 3941 |
| Use of antihypertensive agents | <32 weeks | 1021 | 3320 |
|  | ≥32 weeks | 427 | 3923 |
| Use of magnesiumsulfate | <32 weeks | 1021 | 3320 |
|  | ≥32 weeks | 427 | 3923 |

CPR, cerebroplacental ratio; CTG, cardiotocography; DBP, diastolic blood pressure; DV, ductus venosus; EDF, end-diastolic flow; HELLP, hemolysis elevated liver enzymes low platelets; MCA, middle cerebral artery; PI, pulsatility index; PIV, pulsatility index of veins; UA, umbilical artery.

**Figure S1.** Time sequence of fetal parameters in relation to time before birth stratified according to gestational age at birth per subgroup

*
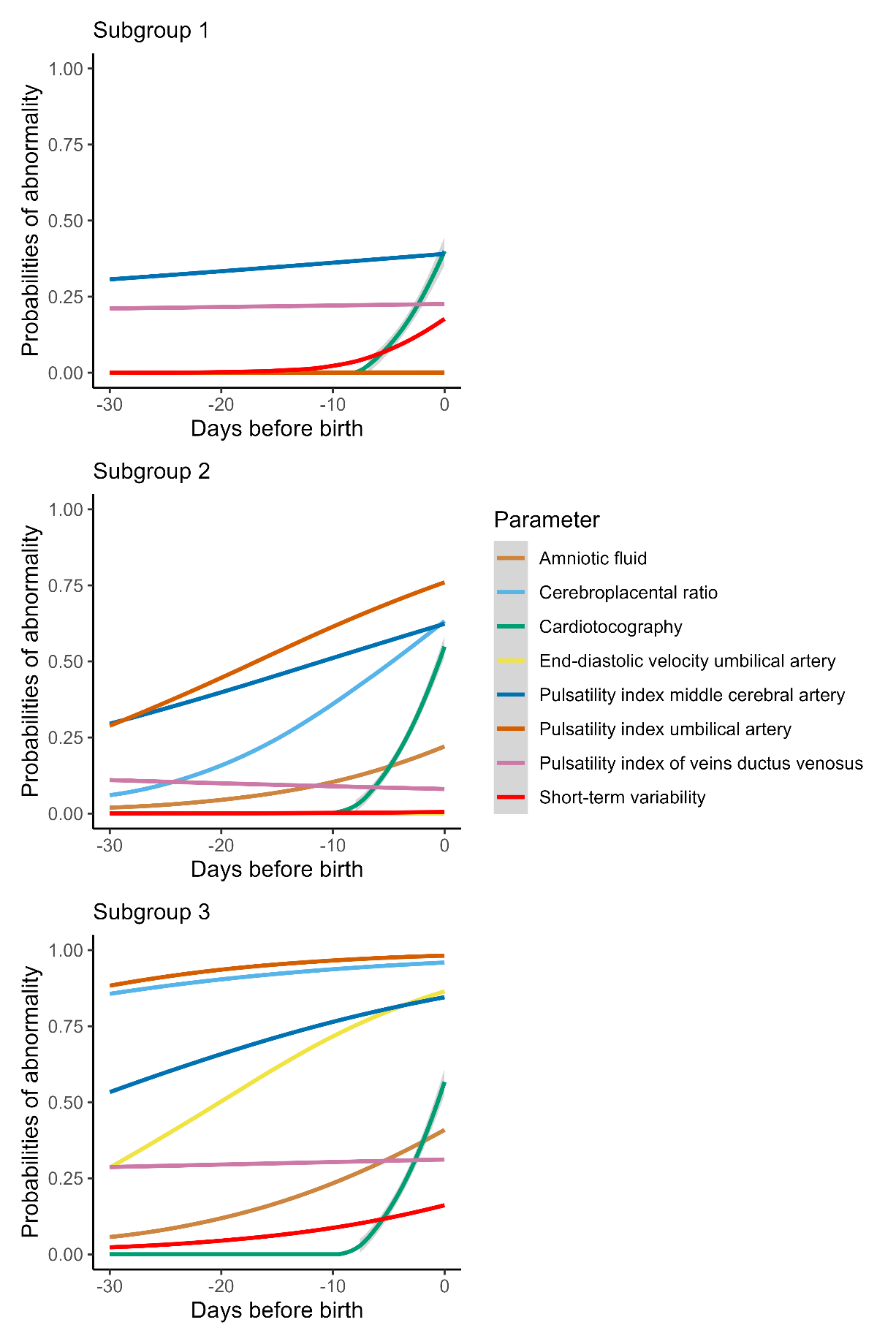
*

**Figure S2.** Time sequence of maternal parameters in relation to time before birth stratified according to gestational age at birth per subgroup

*
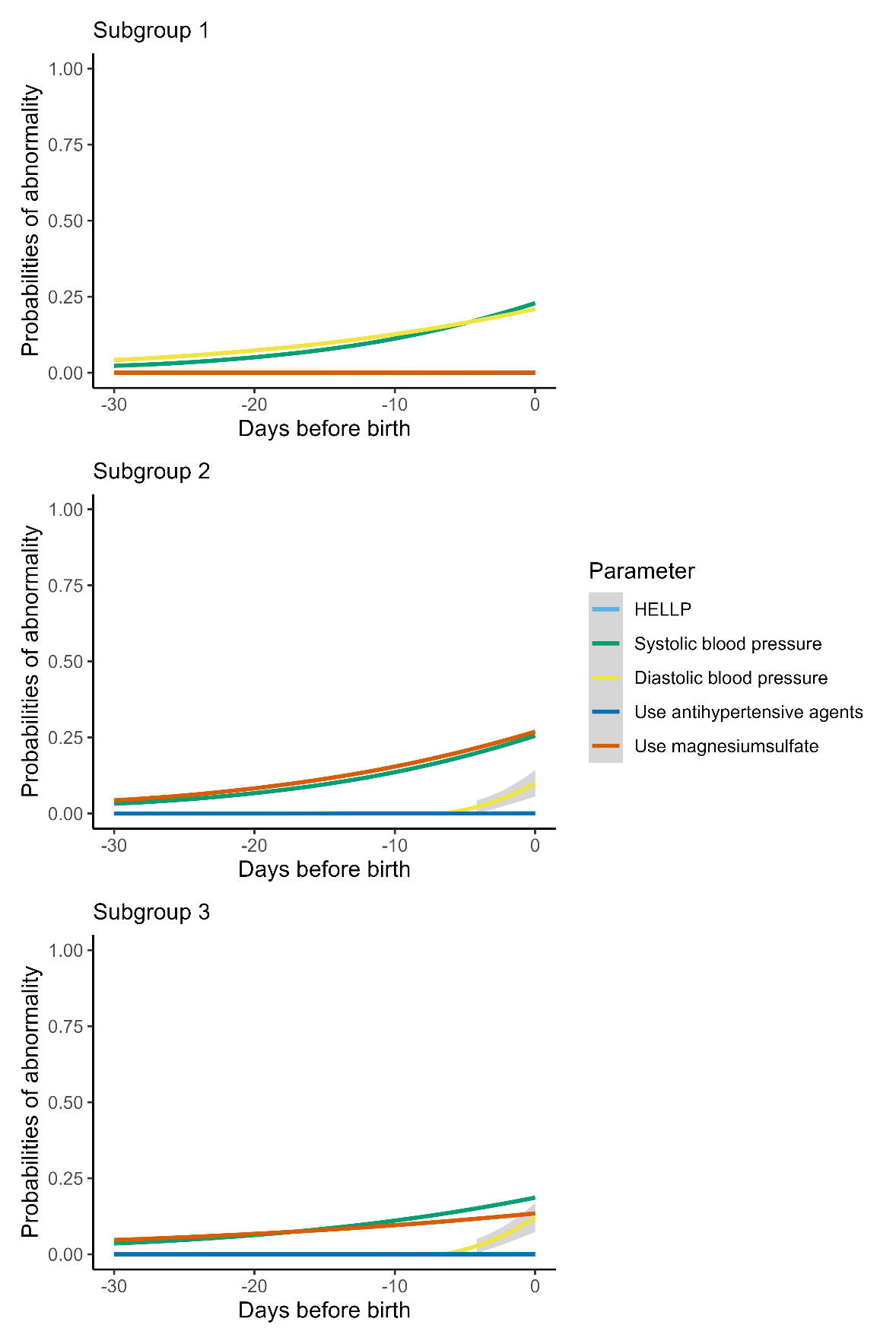
*

*Abbreviations: HELLP, hemolysis elevated liver enzymes low platelets. Figure 2A and 2B describe the time sequence of parameters in the complete group of early-onset FGR pregnancies, 2C and 2D describe the time sequence in patients with early-onset FGR without concomitant PE.*

**File 1.** STROBE Statement—checklist of items that should be included in reports of observational studies

|  | Item No. | Recommendation | Page  No. | Relevant text from manuscript |
| --- | --- | --- | --- | --- |
| **Title and abstract** | 1 | (*a*) Indicate the study’s design with a commonly used term in the title or the abstract | 1 | “a multicenter, retrospective cohort study” |
|  |  | (*b*) Provide in the abstract an informative and balanced summary of what was done and what was found | 2-3 |  |
| Introduction | | | |  |
| Background/rationale | 2 | Explain the scientific background and rationale for the investigation being reported | 4-5 |  |
| Objectives | 3 | State specific objectives, including any prespecified hypotheses | 4-5 | “The aim of this study was to describe the time sequence of changes in fetal and maternal parameters in a large contemporary cohort of early-onset FGR pregnancies. Second, this study aimed to link predefined clusters of early-onset FGR phenotypes to neonatal outcomes to further guide clinical decision-making.” |
| Methods | | | |  |
| Study design | 4 | Present key elements of study design early in the paper | 5 | “In short, a multicenter, retrospective cohort study was performed in six tertiary teaching hospitals in the Netherlands between 2012-2021.” |
| Setting | 5 | Describe the setting, locations, and relevant dates, including periods of recruitment, exposure, follow-up, and data collection | 5-6 | “In short, a multicenter, retrospective cohort study was performed in six tertiary teaching hospitals in the Netherlands between 2012-2021.” |
| Participants | 6 | (*a*) *Cohort study*—Give the eligibility criteria, and the sources and methods of selection of participants. Describe methods of follow-up  *Case-control study*—Give the eligibility criteria, and the sources and methods of case ascertainment and control selection. Give the rationale for the choice of cases and controls  *Cross-sectional study*—Give the eligibility criteria, and the sources and methods of selection of participants | 5 | “To be eligible for inclusion within the OPTICORE cohort patients had to be diagnosed with early-onset FGR in accordance with the consensus-based definition of Gordijn et al.13, it had to be a singleton pregnancy, patients had to opt for active fetal management and had to be ≥18 years of age. Multiple pregnancies, pregnancies diagnosed with a fetal genetic or congenital disorder and patients who indicated that their data or offspring data was not available for scientific purposes were excluded. Within the OPTICORE study, repeated measurements were included at the day of each ultrasound examination.” |
|  |  | (*b*) *Cohort study*—For matched studies, give matching criteria and number of exposed and unexposed  *Case-control study*—For matched studies, give matching criteria and the number of controls per case | NA |  |
| Variables | 7 | Clearly define all outcomes, exposures, predictors, potential confounders, and effect modifiers. Give diagnostic criteria, if applicable | 6 | “For each cluster, gestational age at birth, birthweight, rates of prelabour section, rates of birth <34 weeks and a composite of adverse perinatal outcome (i.e. perinatal or in-hospital mortality, necrotizing enterocolitis ≥2A, moderate or severe bronchopulmonary dysplasia, cystic periventricular leukomalacia, intraventricular hemorrhage grade 3 or venous infarction and/or culture-proven sepsis) were described.” |
| Data sources/ measurement | 8* | For each variable of interest, give sources of data and details of methods of assessment (measurement). Describe comparability of assessment methods if there is more than one group | 4-6 | “Fetal and maternal parameters were collected routinely from diagnosis of early-onset FGR until birth. The frequency of monitoring and availability of parameters depended on the severity of disease in accordance with local monitoring guidelines.” |
| Bias | 9 | Describe any efforts to address potential sources of bias | NA |  |
| Study size | 10 | Explain how the study size was arrived at | NA |  |

Continued on next page

| Quantitative variables | 11 | Explain how quantitative variables were handled in the analyses. If applicable, describe which groupings were chosen and why | 6-7 |  |
| --- | --- | --- | --- | --- |
| Statistical methods | 12 | (*a*) Describe all statistical methods, including those used to control for confounding | 7 |  |
|  |  | (*b*) Describe any methods used to examine subgroups and interactions | NA |  |
|  |  | (*c*) Explain how missing data were addressed | NA |  |
|  |  | (*d*) *Cohort study*—If applicable, explain how loss to follow-up was addressed  *Case-control study*—If applicable, explain how matching of cases and controls was addressed  *Cross-sectional study*—If applicable, describe analytical methods taking account of sampling strategy | NA |  |
|  |  | (*e*) Describe any sensitivity analyses | 7 |  |
| Results | | | | |
| Participants | 13* | (a) Report numbers of individuals at each stage of study—eg numbers potentially eligible, examined for eligibility, confirmed eligible, included in the study, completing follow-up, and analysed | 7 | “A total of 1,453 patients were eligible for inclusion in this post-hoc analysis of the OPTICORE study.” |
|  |  | (b) Give reasons for non-participation at each stage | 7 |  |
|  |  | (c) Consider use of a flow diagram | NA |  |
| Descriptive data | 14* | (a) Give characteristics of study participants (eg demographic, clinical, social) and information on exposures and potential confounders | 7-8 |  |
|  |  | (b) Indicate number of participants with missing data for each variable of interest | NA |  |
|  |  | (c) *Cohort study*—Summarise follow-up time (eg, average and total amount) | 8-9 | Figure 1-3 |
| Outcome data | 15* | *Cohort study*—Report numbers of outcome events or summary measures over time | 9, 18 |  |
|  |  | *Case-control study—*Report numbers in each exposure category, or summary measures of exposure | NA |  |
|  |  | *Cross-sectional study—*Report numbers of outcome events or summary measures | NA |  |
| Main results | 16 | (*a*) Give unadjusted estimates and, if applicable, confounder-adjusted estimates and their precision (eg, 95% confidence interval). Make clear which confounders were adjusted for and why they were included | 9, 18 | Table 3 |
|  |  | (*b*) Report category boundaries when continuous variables were categorized | 18 | Table 3 |
|  |  | (*c*) If relevant, consider translating estimates of relative risk into absolute risk for a meaningful time period | NA |  |

Continued on next page

| Other analyses | 17 | Report other analyses done—eg analyses of subgroups and interactions, and sensitivity analyses | 8-9 | Figure 1-2 |
| --- | --- | --- | --- | --- |
| Discussion | | | | |
| Key results | 18 | Summarise key results with reference to study objectives | 10 | “This study provides the time sequences of changes in fetal and maternal parameters in relation to time before birth in early-onset FGR pregnancies. In the last days preceding birth, the end-diastolic velocity in the umbilical artery and visual CTG assessment had an increased probability to become abnormal. With regard to maternal parameters, an increased use of antihypertensive agent(s) and rate of HELLP diagnosis was seen the last days preceding birth in the group that delivered before 32 weeks. Furthermore, we observed a clear association between the predefined early-onset FGR phenotypes and neonatal outcomes, which could make this subdivision useful for clinical management.” |
| Limitations | 19 | Discuss limitations of the study, taking into account sources of potential bias or imprecision. Discuss both direction and magnitude of any potential bias | 12-13 | “Limitations were that 1) due to feasibility reasons measurements were only included on the day of every ultrasound scan, while for some measurements (e.g. cardiotocography, blood pressure) more measurements would have been available but were left out of the dataset. Inclusion of these daily measurements might have resulted in a even more precise time sequence for these parameters; 2) the predicted probabilities of CTG abnormalities might be biased by indication, since this was the trigger to indicate birth. However, this does reflect clinical practice, as international guidelines on FGR advise to strive for delivery when repeated decelerations on CTG occur; 3) the monitoring frequency differed between patients, mostly depending on disease severity, which could have influenced the accuracy of the time sequence further, although this also reflects current clinical practice; 4) due to regularization, model predictions are pulled towards the mean for better generalizability, resulting in less extreme probabilities compared to observed values; 5) while this study provides valuable insights into the progression of parameters over time, it focuses on early-onset FGR patients as a group, making it unsuitable for use in individual patients.” |
| Interpretation | 20 | Give a cautious overall interpretation of results considering objectives, limitations, multiplicity of analyses, results from similar studies, and other relevant evidence | 10-13 |  |
| Generalisability | 21 | Discuss the generalisability (external validity) of the study results | 10-13 |  |
| Other information | |  | | |
| Funding | 22 | Give the source of funding and the role of the funders for the present study and, if applicable, for the original study on which the present article is based | 14 | “This research was funded by SGS Achmea (2021381). The funding source had no involvement in the study design; the collection, analysis and interpretation of data; in the writing of the report; and in the decision to submit the article for publication.” |

*Give information separately for cases and controls in case-control studies and, if applicable, for exposed and unexposed groups in cohort and cross-sectional studies.

**Note:** An Explanation and Elaboration article discusses each checklist item and gives methodological background and published examples of transparent reporting. The STROBE checklist is best used in conjunction with this article (freely available on the Web sites of PLoS Medicine at http://www.plosmedicine.org/, Annals of Internal Medicine at http://www.annals.org/, and Epidemiology at http://www.epidem.com/). Information on the STROBE Initiative is available at www.strobe-statement.org.

**REFERENCES**

1. Khalil A, Sotiriadis A, D’Antonio F, Da Silva Costa F, Odibo A, Prefumo F, et al. ISUOG Practice Guidelines: performance of third-trimester obstetric ultrasound scan. Ultrasound in Obstetrics and Gynecology. 2024 Jan 1;63(1):131–47.

2. Ayres‐de‐Campos D, Spong CY, Chandraharan E. FIGO consensus guidelines on intrapartum fetal monitoring: Cardiotocography. International Journal of Gynecology & Obstetrics. 2015 Oct 30;131(1):13–24.

3. Lees C, Marlow N, Arabin B, Bilardo CM, Brezinka C, Derks JB, et al. Perinatal morbidity and mortality in early-onset fetal growth restriction: Cohort outcomes of the trial of randomized umbilical and fetal flow in Europe (TRUFFLE). Ultrasound in Obstetrics and Gynecology. 2013;42(4):400–8.
